# Exploring relationships between conflict intensity, forced displacement, and healthcare attacks: a retrospective analysis from Syria, 2016–2022

**DOI:** 10.1186/s13031-024-00630-4

**Published:** 2024-11-21

**Authors:** Maia C. Tarnas, Mohamed Hamze, Bachir Tajaldin, Richard Sullivan, Daniel M. Parker, Aula Abbara

**Affiliations:** 1 Department of Population Health and Disease Prevention, University of California, Irvine, USA; 2 Syrian American Medical Society, Washington, DC USA; 3 King’s College London, London, UK; 4 Department of Epidemiology and Biostatistics, University of California, Irvine, USA; 5 Imperial College, London, UK

**Keywords:** Syria; attacks on healthcare, International humanitarian law, Displacement

## Abstract

**Introduction:**

Attacks on healthcare have been committed throughout the Syrian conflict in violation of International Humanitarian Law (IHL), contributing to the devastation of the country’s healthcare system. The conflict has also forcibly displaced over half of Syria’s pre-conflict population, 7.2 million of whom are internally displaced. In this retrospective analysis, we aim to assess the relationships between (1) healthcare attacks and general conflict and (2) healthcare attacks and forced displacement between 2016 and 2022.

**Methods:**

Data on healthcare attacks, conflict events, and displacement were extracted from the Syrian American Medical Society (SAMS), Uppsala Conflict Data Project, and OCHA Türkiye, respectively. The analysis addresses three research questions: the associations between (1) healthcare attacks and conflict events, (2) healthcare attacks and conflict events in the week after an attack on a healthcare facility, and (3) healthcare attacks and forced displacement. For each, we used generalized additive models with a negative binomial distribution that also accounted for spatial and temporal factors.

**Results:**

SAMS recorded a total of 541 attack events, comprising 650 attack rounds over 235 facilities between 2016 and 2022. Conflict events were significantly associated with healthcare attacks in the same week (IRR: 1.14, 95% CI 1.12–1.17), and healthcare attacks in one week were associated with a maximum of 1.44 greater risk (95% CI 1.08–1.91) of conflict events in the following week, even when accounting for general conflict levels in the previous weeks. Healthcare attacks were also significantly associated with increased displacement up to three months following the attacks.

**Discussion:**

We find that healthcare facilities are not avoided during conflict (as obliged under IHL), and that healthcare attacks significantly precede an escalation of general conflict in the same area. Healthcare attacks are also significantly associated with displacement for months following the attacks, even when accounting for conflict levels. Based on these findings, we present a framework outlining one pathway through which healthcare attacks may contribute to larger conflict tactics. Our findings highlight the critical role of healthcare infrastructure in conflict and reaffirm calls to hold perpetrators of these attacks accountable.

**Supplementary Information:**

The online version contains supplementary material available at 10.1186/s13031-024-00630-4.

## Introduction

Attacks on healthcare, despite violating International Humanitarian Law (IHL), are becoming the new global ‘norm.’ Syria’s conflict, which escalated after peaceful demonstrations in March 2011, has experienced among the most severe and extensive healthcare attacks. Physicians for Human Rights (PHR) documented 604 healthcare attacks and the killing of 949 healthcare workers, many of whom were kidnapped or detained before their deaths, between 2011 and March 2024 [1]. Such healthcare attacks during the Syrian conflict have taken many forms. From early in the conflict, the provision of care to individuals deemed to be in opposition to the government or living in opposition-held areas was effectively criminalized which led to the targeting, torture, arrest, and killing of healthcare workers [2, 3]. This contributed to a mass exodus of healthcare workers from the country [4]. For those who stayed, many cite long-lasting and deep impacts on their wellbeing as a repercussion of the attacks [3]. Armed assaults, looting of healthcare facilities, and blocking or obstructing ambulances are also common in Syria. A review of data from the Syrian Network for Human Rights found that nearly 50% of ambulances involved in attacks were heavily damaged or put out of service [5]. Broadly, healthcare attacks have resounding impacts on the health system, including delivery of care and governance, and have reverberating effects on the community’s health [6].

Increasingly, attacks have taken the form of airstrikes on healthcare facilities (including ambulances and mobile clinics); this includes the use of untargeted, wide-area weapons such as barrel and cluster bombs [7]. These aerial attacks—on healthcare [5, 8] and other civilian infrastructure—are the main form of combat used by the governments of Syria and Russia (Syria’s primary ally) [9]. This includes ‘double-tap attacks,’ where one airstrike is closely followed by a second (or multiple), effectively targeting first responders attending the scene of the initial attack [10, 11]. Deliberate attacks against healthcare such as these are explicitly prohibited under IHL, as are indiscriminate attacks that could not or did not discriminate against military and civilian objectives [12, 13]. Moreover, healthcare facilities are granted specific protections under IHL [14, 15]. Such obligations, however, have not prevented warring parties in Syria from committing a staggering number of healthcare attacks. Attacks against healthcare are also part of larger siege warfare tactics seen throughout the conflict that restrict necessary resources, including humanitarian aid, food, and medical care [9, 10]. These tactics are most common in areas under opposition control (such as Eastern Ghouta, Eastern Aleppo, and the city of Homs), which have been subject to repeated targeting of healthcare facilities, sieges, and restricted access to care [16–18].

Healthcare attacks have contributed to the widespread destruction of critical medical infrastructure throughout the conflict and led to large-scale forced displacement [2, 19]. Over the 13 years of conflict, over 7.2 million people have been internally displaced, with an additional 6.5 million individuals forced to flee the country as refugees [20, 21]. Similar devastation has been seen in the healthcare system, which can now not meet the population’s rising health needs. Across the country, an estimated 50% of hospitals are non-functional or partially functional, as are 53% of all public health facilities with areas in northern Syria particularly affected [22]. The situation for healthcare in Syria is set to worsen with the closure of dozens of facilities in 2024 given the loss of interest among donors [23]. As of October 2024, only 26.5% of UN OCHA’s Humanitarian Response Plan has been funded, leaving a shortfall of 2.99 billion USD from the international community [24].

Several studies have attempted to understand the impact of healthcare attacks in Syria, and more have worked to quantify the number of events that have occurred. Determining an exact number of attacks has been difficult given variations in reporting and verification methodologies [25]. For example, PHR uses media coverage and other open sources to identify attacks, whereas the Syrian American Medical Society (SAMS) uses first-hand data collection [25]. This variation has led to some studies using a single source of attacks [25], and others attempting to combine several datasets [2]. Each methodology has its limitations, and efforts have been made to create a more standardized reporting system or refine existing ones [25, 26]. Studies have found that healthcare attacks can be used as an indicator of civilian violence in Syria and have also noted impacts of these attacks on the health system, delivery of care, workforce retention, and psychological wellbeing [3, 6, 9, 27]. However, there have been few attempts to quantitatively assess the role of healthcare attacks in larger conflict tactics and dynamics, including population displacement. This paper aims to assess the relationships between 1) healthcare attacks and general conflict, and 2) healthcare attacks and displacement.

## Methods

This retrospective analysis (2016–2022) was split into two parts, the first of which assessed the relationship between healthcare attacks and general conflict levels, and the second of which analyzed the association between healthcare attacks and population displacement. In the analyses, we used data on healthcare attacks collected by SAMS, one of the primary nongovernmental organizations (NGOs) that provides healthcare in areas of Syria affected by conflict. In addition to providing medical care, SAMS has documented attacks on its healthcare facilities and those of other NGOs since 2014. These facilities include hospitals, primary care centers, ambulance networks, and mobile clinics throughout Syria, though most are located in the northwest (most of Idlib and parts of Aleppo governorate) given the heavy conflict experienced there [28]. We included 22 districts within eight governorates (Aleppo, Damascus, Dara, Hama, Hassakeh, Homs, Idlib, and Rural Damascus). Most of the districts were in Idlib (n = 5) and Aleppo (n = 5) governorates.

### Healthcare attacks data

SAMS provided its database of documented healthcare attacks on SAMS facilities and those of other NGOs. The attacks include several modalities such as aerial attacks (including missiles, cluster bombs, barrel bombs, and chemical attacks), improvised explosive devices, theft, assault and arrest within a healthcare facility, and shootings. Attacks on healthcare workers that occurred outside of the healthcare facility were excluded, as were attacks with insufficient detail. The methodology SAMS uses to document each attack has been reported elsewhere [29]. Briefly, each healthcare facility has a security focal point responsible for contacting SAMS following an attack; SAMS then completes an incident report form detailing the attack and, when accessible, sends a field media team to document the attack through photographs, videos, and CCTV footage. In 2021, SAMS developed a database of these reports following thorough expert review and harmonization. When multiple rounds of attacks occur on the same facility, on the same day, and using the same modality, SAMS records a single attack event with multiple rounds. For example, an attack event on February 20, 2018, on Arbin Hospital consisted of 10 rounds of attacks. SAMS records this as one attack event with 10 attack rounds. Attacks that occur more than 24 h after the initial report are considered a separate attack event. To capture the magnitude of each attack event, we used the number of attack rounds (i.e., 10 for the Arbin Hospital example) in our analyses. SAMS also reports on facility damage (assessed visually on site and using available documentation), facility status following the attack, and a number of direct injuries and deaths.

We chose to use SAMS data over other databases (such as PHR or WHO’s Surveillance System for Attacks on Health Care (SSA)) or combining databases for several reasons. Each reporting system uses different methodologies, and each has its limitations. There is likely some overlap between the datasets and they are unlikely to be completely independent, meaning that calculating a simple total of events is difficult. Reporting in SSA begins in 2018, which would have limited our time series. The SAMS dataset captures details such as the number of attack rounds, facility damage, facility status following the attack, and direct injuries and deaths that other datasets do not capture systematically or publicly report. Importantly, datasets that rely on secondary open sources, such as PHR, may be more likely to miss ‘smaller’ events such as theft or armed assault that are less likely to be reported in the media but are nevertheless relevant attacks. Healthcare attacks are underreported in this conflict [25], and thus we opted for the database that was more likely to capture a broad scope of events through its direct field reporting methodologies and that retained detailed incident reports for further verification.

### General conflict and displacement data

We used data from the Uppsala Conflict Data Program (UCDP), which has been used similarly elsewhere [30–32], on individual conflict events throughout Syria to measure the level of general conflict. Data were aggregated at each model’s temporal and spatial scale, and we subtracted the number of healthcare attack events from the total number of conflict events during the same period to conservatively capture non-healthcare attack events. When subtracting, the type of event in UCDP was not considered and the number of healthcare attack events was used (rather than attack rounds) to align with UCDP reporting methods. We extracted internal displacement data from OCHA Türkiye via Humanitarian Data Exchange, which are available for each governorate on a monthly scale beginning in January 2016.

### Model specification

We applied three generalized additive models (GAMs) with negative binomial distributions in this study, each of which included data from January 2016 to December 2022. The first assessed the association between weekly healthcare attacks (as the outcome variable) and general conflict at the district level. We accounted for the week of study and included an interaction term between each district centroid’s latitude and longitude to account for baseline spatial factors relevant to the outcome. We initially included a binary variable for Ramadan as there is some anecdotal evidence that attacks increase during this period but excluded it in the final model as it was not significant and did not improve model fit. District area (in km^2^) was also excluded as it had high concurvity with the centroid interaction term.

The second model assessed whether healthcare attacks in one week were associated with conflict events in the following week (i.e., whether healthcare attacks significantly preceded general conflict events). In this model, healthcare attacks were a predictor variable, which we specified categorically to preserve its nonlinear nature while increasing its interpretability. Non-zero attack categories were created using tertiles, and these were measured against a reference category of zero attacks. We also accounted for temporal and spatial factors, in addition to including autoregressive terms for the number of general conflict events in the prior 1, 2, 3, and 4 weeks.

Lastly, the third model assessed the relationship between monthly healthcare attacks and displacement (outcome variable) at the governorate level. We log-transformed the conflict event data in this model to account for skewness at the month-level aggregation. As with the second model, we modeled the healthcare attacks variable categorically using non-zero attack tertiles that were measured against a reference category of zero attacks. In the appendix (p. 5), we include a model that accounts for the number of healthcare attacks in the prior 1, 2, and 3 months; we chose to not include these in the final model based on model fit statistics and because they did not meaningfully change the model outputs. The final model included the month of study, a categorical governorate variable, and autoregressive terms for displacement in the prior 1, 2, and 3 months to account for potential effects of prior displacement. We ran the model with the number of healthcare attacks lagged by 0, 1, 3, and 6 months to assess the relationship over time. All statistical analyses were done in R (version 4.0.4), and the equations and model fit statistics for each model are in the appendix (p. 1 and 6).

## Results

**Fig. 1:**
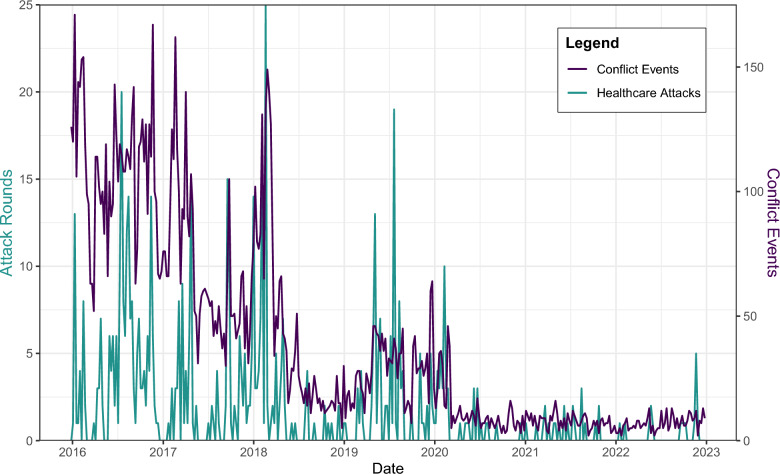
Graph of healthcare attack rounds and conflict events from 2016 to 2022

**Fig. 2:**
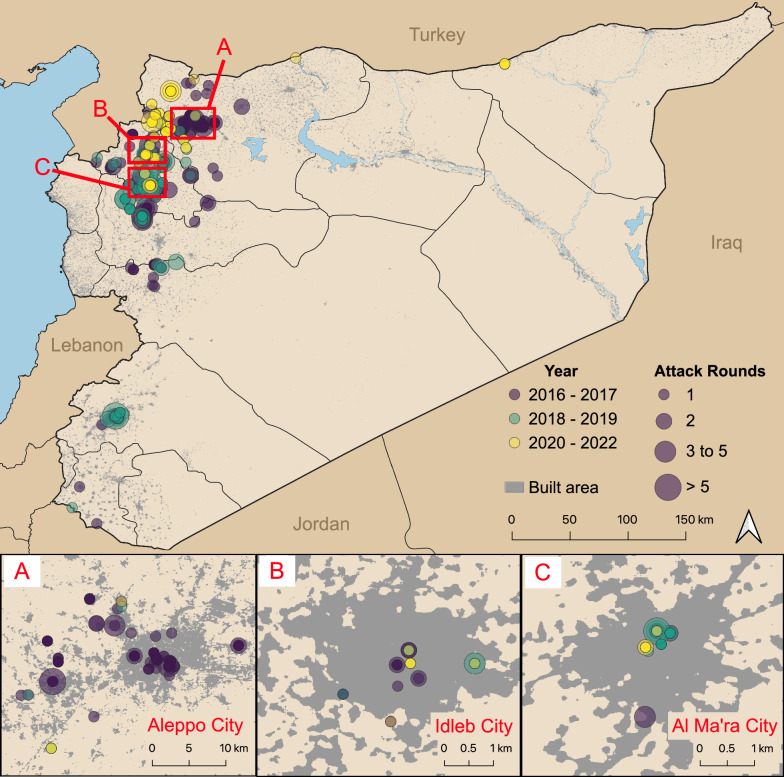
Map of the study area. The three highlighted areas are those that faced the highest number of attack rounds over the study period. Aleppo City is in Jebel Saman district, Idlib City in Idlib district, and Al Ma’ra City in Al Ma’ra district. Built area indicates areas with buildings, housing, roads, or other infrastructure. Map created using QGIS version 3.22.10

Between 2016 and 2022, a total of 541 healthcare attack events were recorded, comprising 650 attack rounds across 208 facilities (Fig. 1). Attack events were most common in Jebel Saman, Aleppo (n = 166, 31%), Al Ma’ra, Idlib (n = 94, 17%), and Idlib, Idlib (n = 85, 16%) (Fig. 2). Maarrat Al Nu'man National Hospital (Al Ma’ra) faced the most attack rounds (n = 27), followed by Cave Hospital Kafr Zeita (n = 21, Muhradah, Hama). Attack events peaked in 2016 (n = 188). Ten percent (n = 55) of attack events had multiple rounds, and most attacks occurred during daylight hours (08:00–18:00). A total of 983 direct injuries and 384 direct deaths were recorded.

General hospitals were most frequently targeted (n = 227 attack events, 42%), followed by primary care centers (n = 122, 23%) and specialized hospitals or facilities (n = 91, 17%). Attacks most frequently caused facilities to close temporarily or temporarily suspend operations (n = 202 attack events, 37%), remain fully open (n = 116, 21%), or remain partially open (n = 91, 17%). Thirty-six facilities were closed permanently and 10 were moved to different locations. Air attacks (using missiles, cluster bombs, barrel bombs, and chemical weapons) were the most common type of event (n = 456, 84%) followed by ground assaults (n = 49, 9%). Table 1 shows the distribution of attacks by governorate, year, and facility damage. Facility damage reflects the amount of physical damage the facility sustained and includes mild (damage requiring minor repairs), moderate (damage that affects larger areas and requires more serious repairs but leaves main areas partially accessible), and severe (significant damage that affects most areas and leaves them inaccessible, requiring extensive maintenance work). This does not necessarily align with whether the facility remained open or closed following the attack. For example, after an attack with minor damage, a facility may have still closed permanently if the level of surrounding violence was deemed too unsafe to continue operations.

**Table 1:** Number of attack events and attack rounds per year and governorate by facility damage

Governorate	Facility damage attack events (Rounds)				
	Mild	Moderate	Severe	Unknown	Total
Idlib	160 (186)	24 (34)	54 (77)	7 (7)	245 (304)
2016–2017	51 (63)	11 (14)	30 (44)	2 (2)	94 (123)
2018–2019	75 (88)	12 (16)	21 (30)	0	108 (134)
2020–2022	34 (35)	1 (4)	3 (3)	5 (5)	43 (47)
Aleppo	101 (103)	11 (13)	61 (81)	7 (7)	180 (204)
2016–2017	51 (52)	8 (8)	56 (75)	6 (6)	121 (141)
2018–2019	25 (25)	0	3 (3)	0	28 (28)
2020–2022	25 (26)	3 (5)	2 (3)	1 (1)	31 (35)
Hama	23 (35)	3 (3)	17 (17)	2 (2)	45 (57)
2016–2017	16 (26)	3 (3)	10 (10)	0	29 (39)
2018–2019	7 (9)	0	7 (7)	2 (2)	16 (18)
2020–2022	0	0	0	0	0
Rural Damascus	16 (25)	4 (4)	21 (23)	0	41 (52)
2016–2017	11 (11)	2 (2)	8 (9)	0	21 (22)
2018–2019	5 (14)	2 (2)	13 (14)	0	20 (30)
2020–2022	0	0	0	0	0
Homs	13 (13)	3 (5)	4 (4)	0	20 (22)
2016–2017	12 (12)	0	4 (4)	0	16 (16)
2018–2019	1 (1)	3 (5)	0	0	4 (6)
2020–2022	0	0	0	0	0
Dara	2 (2)	1 (1)	0	0	3 (3)
2016–2017	1 (1)	1 (1)	0	0	2 (2)
2018–2019	1 (1)	0	0	0	1 (1)
2020–2022	0	0	0	0	0
Al-Hassakeh	4 (4)	0	0	0	4 (4)
2016–2017	0	0	0	0	0
2018–2019	0	0	0	0	0
2020–2022	4 (4)	0	0	0	4 (4)
Damascus	2 (3)	1 (1)	0	0	3 (4)
2016–2017	1 (1)	1 (1)	0	0	2 (2)
2018–2019	1 (2)	0	0	0	1 (2)
2020–2022	0	0	0	0	0
Total	321 (371)	47 (61)	157 (202)	16 (16)	541 (650)

### Association between healthcare attacks and conflict events

Conflict events were significantly associated with healthcare attacks in the same week (IRR: 1.14, 95% CI: 1.12—1.17, p < 0.0001). We also found that across most levels, healthcare attacks were associated with increased risk of conflict events in the week following the attack(s), even when accounting for the number of conflict events in previous weeks. Moreover, healthcare attacks generally showed a stronger association with conflict events in the following week than did the number of previous conflict events, as shown in Table 2. Spline outputs for each of these models are shown in the appendix (p. 2).

**Table 2:** Incidence rate ratios for the association between healthcare attacks in one week and conflict events in the following week

		IRR (95% CI)	*p*-value
Number of healthcare attack rounds in previous week (t–1 week)	None	Comparison	
	1	1.37 (1.16–1.61)	0.0003
	2	1.44 (1.08–1.91)	0.0113
	3 +	0.84 (0.64–1.10)	0.1927
Conflict events	t–1 week	1.10 (1.09–1.11)	< 0.0001
	t–2 week	1.03 (1.02–1.05)	< 0.0001
	t–3 week	1.02 (1.01–1.03)	0.0002
	t–4 week	1.02 (1.01–1.03)	< 0.0001

### Association between healthcare attacks and displacement

Healthcare attacks were significantly associated with displacement; this association was still present three months after the healthcare attacks (Table 3). Four or more healthcare attacks in one month were associated with over double the risk of displacement in that same month (95% CI 1.71–3.53, *p* < 0.0001). In governorates with one healthcare attack, significant displacement began one month later (1.35, 95% CI 1.01–1.81, *p* = 0.02). These associations were no longer significant six months following the healthcare attacks. In each of these models, displacement above approximately 8000 individuals in the prior month was positively associated with displacement in the current month; displacement in the prior two and three months was not significant (appendix p. 3). The outputs for the six-month lag, as well as the month spline functions can be found in the appendix (p. 3–4).

**Table 3:** Incidence rate ratios for the association between healthcare attacks and displacement

	Covariate	No lag		1 Month lag		3 Month lag	
		IRR (95% CI)	*p*-value	IRR (95% CI)	*p*-value	IRR (95% CI)	*p*-value
Healthcare attacks	None	Comparison					
	1	0.91 (0.69–1.21)	0.5291	1.35 (1.01–1.81)	0.0177	1.52 (1.13–2.04)	0.0053
	2–3	0.89 (0.63–1.26)	0.5133	0.89 (0.63–1.27)	0.6613	0.92 (0.65–1.31)	0.6514
	4 +	2.46 (1.71–3.53)	< 0.0001	1.14 (0.78–1.66)	0.1065	0.84 (0.58–1.20)	0.3405
Conflict events		1.41 (1.26–1.57)	< 0.0001	1.48 (1.33–1.66)	< 0.0001	1.49 (1.33–1.65)	< 0.0001
Governorate	Rural Damascus	Comparison					
	Idlib	3.27 (2.00–5.36)	< 0.0001	3.85 (2.34–6.33)	< 0.0001	4.40 (2.68–7.22)	< 0.0001
	Aleppo	3.14 (1.97–5.03)	< 0.0001	3.23 (2.01–5.20)	< 0.0001	3.71 (2.32–5.94)	< 0.0001
	Al-Hassakeh	1.88 (1.28–2.75)	0.0012	1.92 (1.30–2.84)	< 0.0001	2.04 (1.38–3.00)	0.0003
	Dara	1.33 (0.86–2.05)	0.2040	1.29 (0.82–2.01)	0.0010	1.34 (0.86–2.08)	0.1974
	Damascus	0.87 (0.53–1.44)	0.5935	0.83 (0.50–1.38)	0.0439	0.84 (0.50–1.39)	0.4907
	Hama	0.75 (0.52–1.10)	0.1401	0.86 (0.58–1.25)	0.0010	0.87 (0.60–1.28)	0.4834
	Homs	0.48 (0.33–0.72)	0.0003	0.47 (0.32–0.71)	< 0.0001	0.49 (0.33–0.73)	0.0004
AIC		8,745.30		8,771.07		8,764.69	

AIC is the Akaike Information Criterion, where a lower value indicates a better model fit. The conflict events variable is log-transformed.

## Discussion

We find that healthcare attacks play an important role in larger conflict dynamics within the Syrian conflict. This is seen in three primary ways: (1) healthcare facilities are not avoided during the conflict, (2) healthcare attacks significantly precede other conflict events, and (3) healthcare attacks are associated with increased population displacement. Healthcare attacks in one week were generally more strongly associated with conflict events in the following week than recent conflict levels were, suggesting that healthcare attacks may be a better predictor of future conflict than prior conflict levels. These findings align with previous work that discusses the use of healthcare attacks by conflict parties prior to other violent action, such as sieges, as a military tactic [9, 16, 17]. We did find, however, that this relationship was no longer significant at 3 or more healthcare attacks in the prior week. The reasons for this are unclear and thus we can only speculate. Based on SAMS records, perpetrators appear to target strategically important and well-fortified facilities with several attack rounds throughout conflict. Other facilities that are less fortified, and thus may sustain damage more easily, may be targeted with fewer attack rounds as part of an incursion strategy (which includes psychological intimidation). By attacking healthcare first, warring parties can reduce individuals’ ability to seek care, which can in turn compound the community’s vulnerability through reduced access, increasing disease incidence, and unmet care needs. From the perspective of the perpetrator, conflict actions are more likely to be successful when committed on a vulnerable population unable to seek care for both new ailments resulting from conflict and existing conditions.

Our findings also indicate that conflict parties do not appear to be meaningfully avoiding healthcare facilities during conflict, in clear violation of IHL [13–15]. This could be through indiscriminate attacks, where healthcare was not specifically targeted but was nonetheless impacted, or through deliberate attacks on healthcare facilities. Whether these attacks were intentional does not change their illegality under IHL’s provision to prohibit attacks—deliberate or indiscriminate—on healthcare. However, almost 10% of attack events consisted of multiple rounds, which exhibits some degree of intentionality to attack an identified location. The alternative finding (an insignificant association between conflict and healthcare attacks) would not have necessarily indicated adherence to IHL, but could have reflected a more strategic and targeted approach to attacking healthcare that followed a pattern separate from that of general conflict. Such strategic targeting would also violate IHL and would be important to consider in future work.

We also found that most recorded attacks were on hospitals (42%), despite there being a higher prevalence of primary care centers in the studied areas. This may suggest that hospitals are strategically targeted as they provide more secondary, emergency, and trauma care, and because they are physically bigger and thus an easier target for pilots when compared with primary care centers. This overall pattern of increased healthcare attacks can be seen in other ongoing conflicts, including in Gaza and Ukraine; as in Syria, these attacks have occurred largely with impunity [33–36]. Ukraine has experienced over 1500 attacks on healthcare workers, facilities, and other medical infrastructure since Russia’s invasion in 2022 [37]; Russia is a key ally to the Government of Syria and has, by one estimate, conducted 42% of air strikes on civilian infrastructure in Syria either alone or in conjunction with the Syrian government [8].

**Fig. 3:**
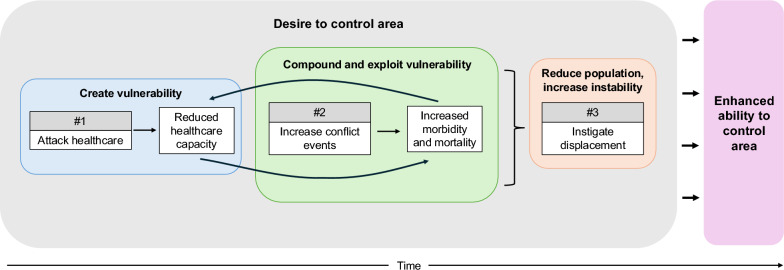
Non-comprehensive framework outlining the role that healthcare attacks may play in larger conflict efforts

Even when controlling for other conflict events, healthcare attacks were significantly associated with displacement. Our results indicate that displacement increases in the same month as four or more healthcare attacks; if there are fewer healthcare attacks, individuals may wait at least one month—but not more than three—before leaving the area. Other work has suggested that healthcare attacks are an important precursor to displacement [9, 10, 38], and the unique devastation and vulnerability that healthcare attacks can create may explain individuals’ motivation to leave the area. Of course, attacks on other critical infrastructure such as that related to water, sanitation, and hygiene (WASH) or education (which are also largely protected under IHL), have occurred throughout the Syrian conflict and likely also affect displacement [8]. Such violence strips individuals of their necessities, including the perception of safety, and is pervasive in its reach. However, these findings suggest that healthcare attacks may be a decisive factor for individuals. Given our findings in aggregate, we propose a framework to explain one potential pathway through which healthcare attacks may play into larger conflict tactics (Fig. 3).

### Limitations

This study has several limitations. We use SAMS data for healthcare attacks, which may not be a complete record of attacks. There is risk of reporting bias in the recording of attacks, where smaller attacks may have gone unrecorded or attacks in certain geographical areas may not be reported because of access challenges. These limitations suggest that the number of attacks used in this study may be an underestimate of the true number of attacks experienced throughout the conflict and that a lack of reported attacks in an area does not necessarily mean that no attacks occurred. Some of the reporting measures—such as the level of facility damage—may be prone to subjectivity by the recorder, though these assessments can be verified by photographs and videos taken by the field team. Recording of attacks by SAMS may also bias towards recording attacks on SAMS facilities rather than on facilities of other NGOs. However, SAMS has established protocols for documenting attacks on other facilities that utilize the same documentation and security teams who record attacks on SAMS facilities; despite this, SAMS may miss documenting some attacks due to safety concerns or access challenges. Again, this indicates that the number of attacks used in this study may be underestimated. There are likewise similar limitations for the recording of general conflict events used in this study. Because we are more interested in relative levels of conflict rather than the precise number of conflict events however, this limitation was unlikely to strongly impact our results.

We use displacement data from OCHA Türkiye, and these too are imperfect. The governorate-level spatial scale of the data prevented us from analyzing the relationship between healthcare attacks and displacement at a smaller spatial scale. Future work that can record displacement and general conflict levels at smaller spatial scales (e.g., sub-districts or neighborhoods) could allow for a more detailed understanding of the attacks’ impacts. There may have also been other factors contributing to displacement, and even general conflict levels, that we were unable to account for in our models. We encourage research that investigates these mechanisms, and especially that which can include a measure of territorial control.

## Conclusion

This study adds to the literature on healthcare attacks during the Syrian conflict by investigating their relationship with general conflict and displacement. We also propose a framework, from our evidence, that offers a way of understanding one role healthcare attacks may take in this conflict. These findings highlight ways in which healthcare attacks may be used strategically as precursors to other conflict events, and how IHL provisions that protect healthcare (and other civilian infrastructure) have been disregarded throughout this conflict. Moreover, we find that even when accounting for conflict levels, healthcare attacks are significantly associated with displacement. Both healthcare attacks and displacement have occurred at an immense scale throughout this conflict and understanding their relationship is important for future advocacy and preparedness work. Further research is needed to explore these relationships at a smaller spatial scale. Our work reinforces the need for greater accountability for perpetrators in this and other conflicts, especially as they continue to engage in these tactics in Syria and elsewhere. Protection of healthcare during conflict is paramount, and flouting this law has devastating results.

## Supplementary Information

Additional file 1.

## Data Availability

Data on conflict events and displacement used in this study are available for public use at https://ucdp.uu.se/country/652 and https://data.humdata.org/dataset/syrian-arab-republic-idp-movements-and-idp-spontaneous-return-movements-data. SAMS healthcare attack data may be available upon reasonable request under a data sharing agreement.

## Abbreviations

GAM: Generalized additive model
IHL: International Humanitarian Law
IRR: Incidence rate ratio
PHR: Physicians for Human Rights
NGO: Nongovernmental organization
OCHA: United Nations Office for the Coordination of Humanitarian Affairs
SAMS: Syrian American Medical Society
SSA: Surveillance System for Attacks on Health Care
UCDP: Uppsala Conflict Data Program
WHO: World Health Organization
