# Supplementary material for: Exploring relationships between conflict intensity, forced displacement, and healthcare attacks: a retrospective analysis from Syria, 2016–2022
Source: Confl Health. 2024 Nov 21;18:70. doi: 10.1186/s13031-024-00630-4 (PMC11580498; doi:10.1186/s13031-024-00630-4)
Supplement: Supplementary file 1 — Additional file 1. [file 13031_2024_630_MOESM1_ESM.docx]

**Supplementary Material**

Contents

Model Equations…………….……….….………………………………………….….………….1

Spline Outputs for Model 1…………………………..……..………………………….………….2

Spline Outputs for Model 2…………………………..……..………………………….………….2

Spline Outputs for Model 3…………………………..……..………………………….………….3

Additional Model 3 Outputs…………………………..……..…………….…………….…......….4

Model 3 with Prior Healthcare Attacks…..…………..……..…………….…………….…………5

Model Fit Statistics…………………………………..……..………………………….….………6

**Model Equations**

Model 1

*healthcare attacks =* $\beta_{1}$*(conflict events) +* $f_{1}$*(week) +* $f_{2}$*(lon,lat)*

Model 2

*conflict events =* $\beta_{1}$*(low attack rounds_t-1_) +* $\beta_{2}$*(med attack rounds_t-1_) +* $\beta_{3}$*(high attack rounds_t-1_) +* $\beta_{4}$*(conflict events_t-1_) +* $\beta_{5}$*(conflict events_t-2_) +* $\beta_{6}$*(conflict events_t-3_) +* $\beta_{7}$*(conflict events_t-4_) +* $f_{1}$*(week) +* $f_{2}$*(lon,lat)*

Where *t* is weeks, $\beta$_1_ – $\beta$_7_ are linear predictors in the models, and *f*_1_ - *f*_2_ are spline functions estimated using restricted maximum likelihood

Model 3

*displacement =* $\beta_{1}$*(low attack rounds_t-x_) +* $\beta_{2}$*(med attack rounds_t-x_) +* $\beta_{3}$*(high attack rounds_t-x_) +* $\beta_{4}$*(conflict events) +* $f_{1}$*(displacement_t-1_) +* $f_{2}$*(displacement_t-2_) +* $f_{3}$*(displacement_t-3_) +* $\beta_{5}$*(Idlib) +* $\beta_{6}$*(Aleppo) +* $\beta_{7}$*(Hassakeh) +* $\beta_{8}$*(Dara) +* $\beta_{9}$*(Damascus) +* $\beta_{10}$*(Hama) +* $\beta_{11}$*(Homs) +* $f_{4}$*(month)*

Where *t* is months, individual models were run with *x* at 0, 1, 3, and 6, $\beta$_1_ - $\beta$_11_ are linear predictors in the models and *f*_1_ – *f*_4_ are spline functions estimated using restricted maximum likelihood. All models were run with a negative binomial distribution.

**Spline Outputs for Model 1**

The spline outputs for Model 1, which measured the association between conflict events and healthcare attacks (outcome variable) in the same week (**Figure S1)**. The latitude and longitude interaction plot (right) indicates risk by geographical area, with reds indicating less risk and yellows indicating higher risk. In this model, the areas of highest risk are in Idlib.

**
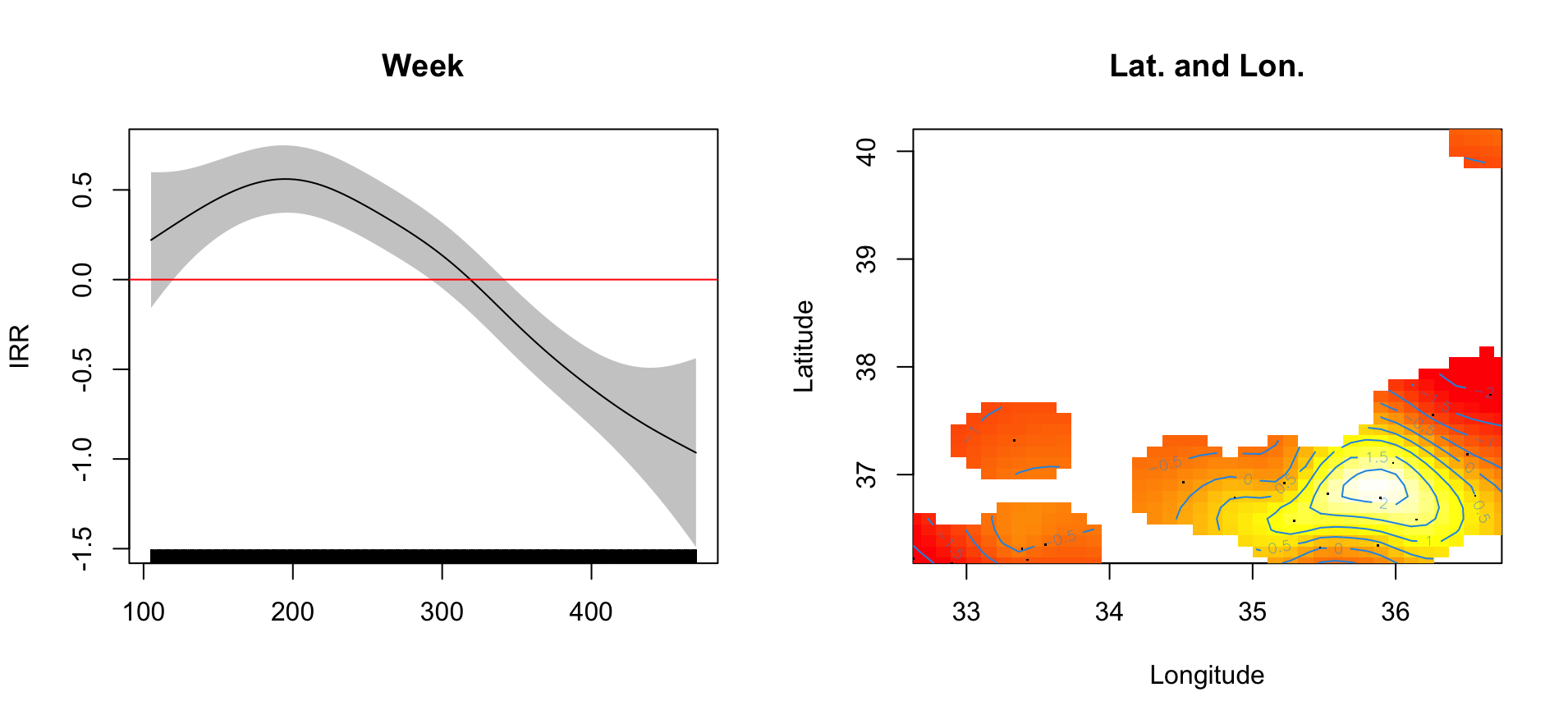
**

**Figure S1:** Spline functions for time and the geographic location (i.e. an interaction spline between latitude and longitude of districts’ centroids) for Model 1. The red line in the time spline can be used to measure significance, where the output is considered significant when the solid line and shaded areas (indicating 95% confidence intervals) are fully above or below the line.

**Spline Outputs for Model 2**

The spline outputs for Model 2, which assessed whether healthcare attacks were associated with conflict events in the following week (**Figure S2)**.

**
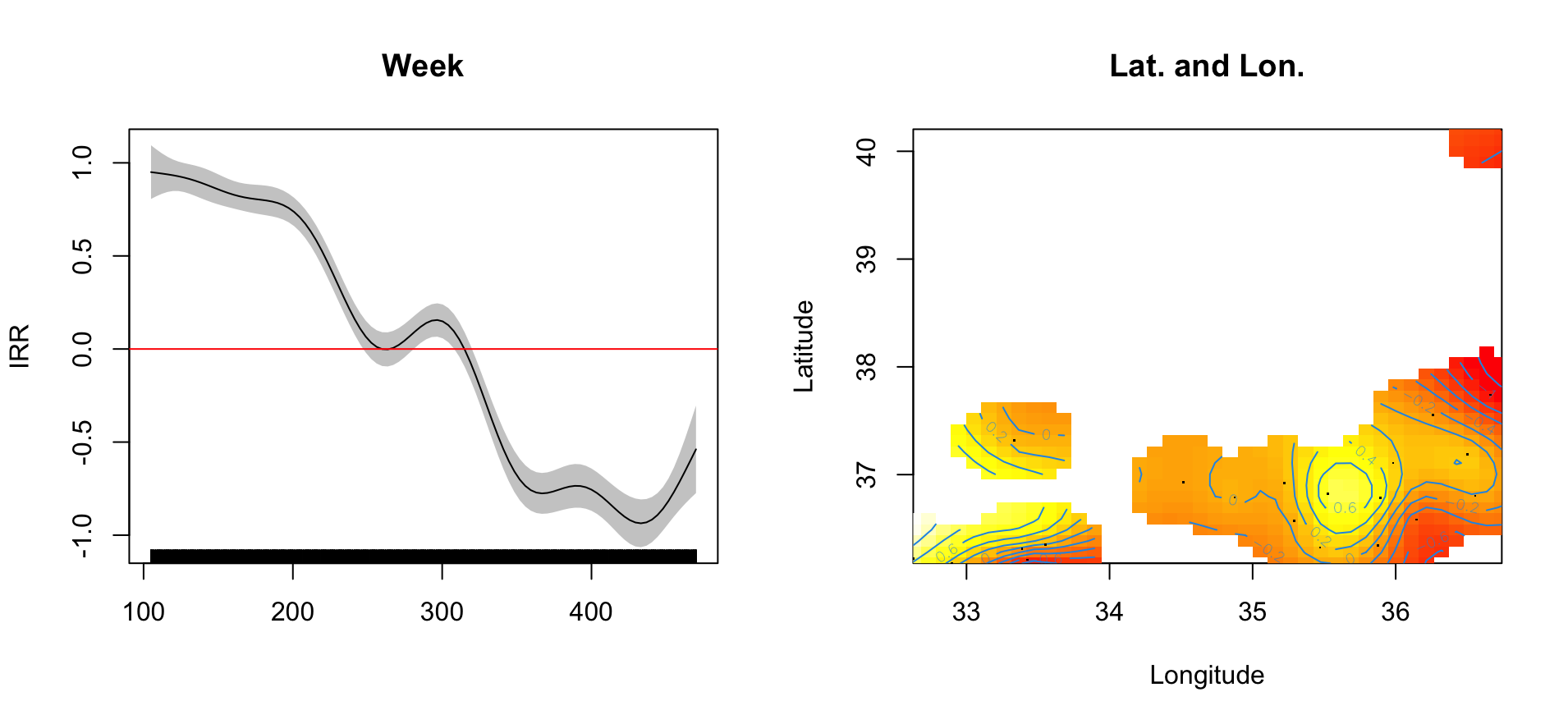
**

**Figure S2:** Spline outputs for week and the interaction between latitude and longitude of districts’ geometric centroids for Model 2.

**Spline Outputs for Model 3**

The spline outputs for Model 3, which measured the association between attacks on healthcare and displacement using autoregressive displacement terms (1, 2, and 3 months), are in **Figure S3**. We ran this model under with the healthcare attack variable lagged 0, 1, 3, and 6 months to assess the association between healthcare attacks and displacement over time.


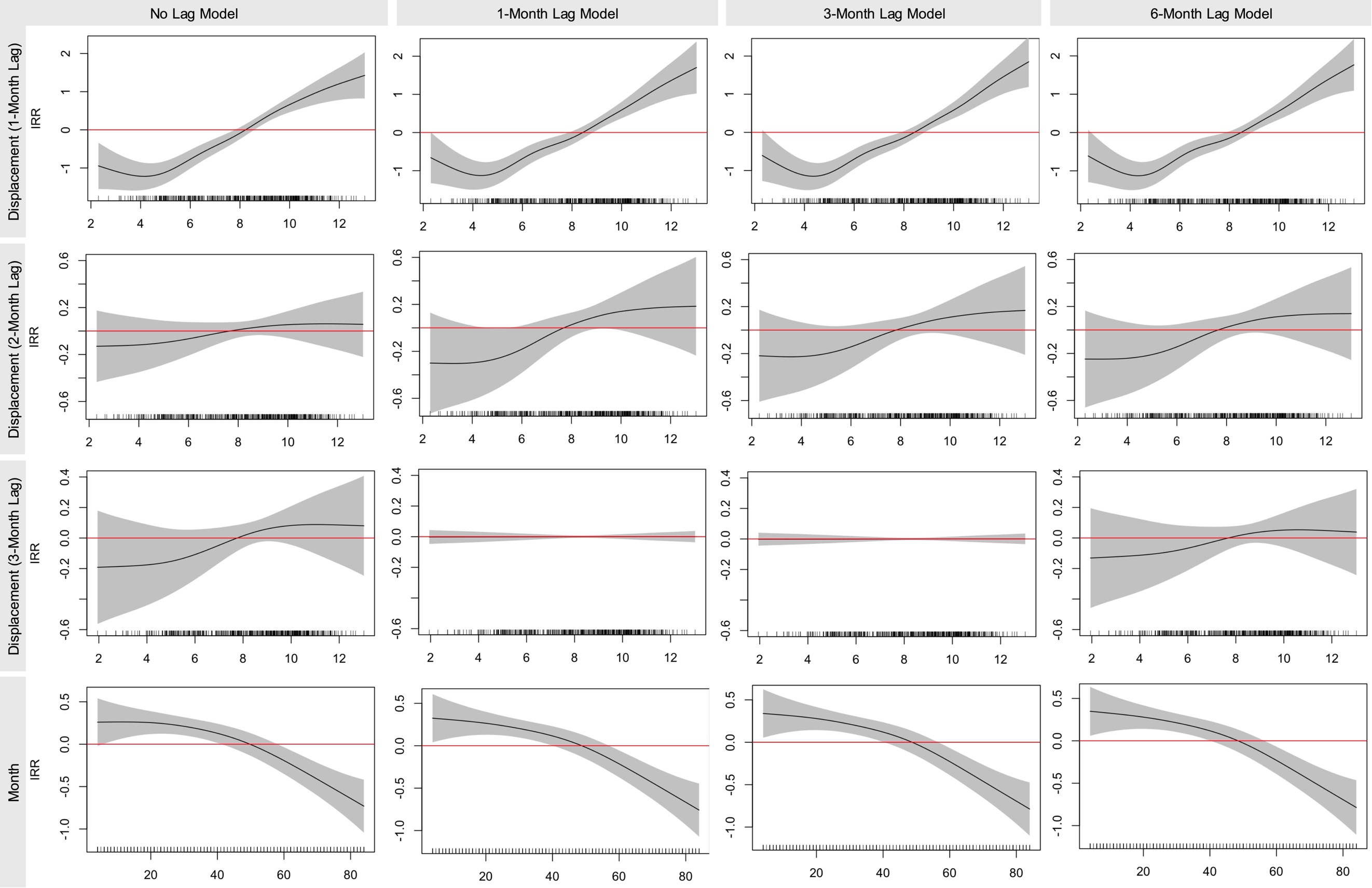


**Figure S3:** Spline outputs for autoregressive displacement terms and month under each model specification. Note that the autoregressive displacement terms are log-transformed.

**Additional Model 3 Outputs**

In addition to the lags shown in the main text, we ran Model 3 with healthcare attacks lagged 6 months. The results are presented in **Table S1**.

|  | **Covariate** | **6 Month Lag** | |
| --- | --- | --- | --- |
|  |  | IRR (95% CI) | p |
| Healthcare attacks | None | Comparison |  |
|  | 1 | 0.85 (0.63 – 1.14) | 0.2782 |
|  | 2 - 3 | 0.90 (0.64 – 1.27) | 0.5568 |
|  | 4+ | 0.89 (0.63 – 1.25) | 0.4940 |
| Conflict events |  | 1.49 (1.34 – 1.66) | <0.0001 |
| Governorate | Rural Damascus | Comparison |  |
|  | Idlib | 4.37 (2.63 – 7.28) | <0.0001 |
|  | Aleppo | 3.68 (2.27 – 5.98) | <0.0001 |
|  | Al-Hassakeh | 1.89 (1.28 – 2.79) | 0.0014 |
|  | Dara | 1.30 (0.83 – 2.02) | 0.2501 |
|  | Damascus | 0.82 (0.49 – 1.36) | 0.4395 |
|  | Hama | 0.94 (0.64 – 1.38) | 0.7435 |
|  | Homs | 0.49 (0.33 – 0.73) | 0.0005 |
| AIC |  | 8,774.28 | |

**Table S1**: Model outputs for healthcare attacks lagged 6 months.

Note that we also ran Model 3 while including the number of individuals directly killed or injured as a result of the attack. This was not significant (IRR: 0.99, 95% CI: 0.98 - 1.0005), indicating that the direct human toll of the attack may not have contributed to individuals’ decision to leave the area.

**Model 3 with Prior Healthcare Attacks**

We ran Model 3 (no lag) while accounting for the number of healthcare attacks in the prior 1, 2, and 3 months to assess whether a history of healthcare attacks affected displacement (**Table S2**). None of the prior attacks were statistically significant, their inclusion did not markedly change the model output, and the model had a slightly higher AIC value than the main model. Based on this, we did not include prior healthcare attacks in the main model.

|  | **Covariate** | | **IRR (95% CI)** | **p** |
| --- | --- | --- | --- | --- |
| Healthcare attacks | Same month | None | Comparison |  |
|  |  | 1 | 0.96 (0.71 – 1.28) | 0.7658 |
|  |  | 2 - 3 | 0.94 (0.66 – 1.34) | 0.7156 |
|  |  | 4+ | 2.33 (1.59 – 3.42) | <0.0001 |
|  | t – 1 month | None | Comparison |  |
|  |  | 1 | 1.07 (0.80 – 1.43) | 0.6629 |
|  |  | 2 - 3 | 0.74 (0.52 – 1.07) | 0.1117 |
|  |  | 4+ | 1.06 (0.71 – 1.57) | 0.7829 |
|  | t – 2 months | None | Comparison |  |
|  |  | 1 | 1.20 (0.89 – 1.61) | 0.2307 |
|  |  | 2 - 3 | 0.94 (0.65 – 1.35) | 0.7250 |
|  |  | 4+ | 0.82 (0.55 – 1.23) | 0.3400 |
|  | t – 3 months | None | Comparison |  |
|  |  | 1 | 1.22 (0.90 – 1.64) | 0.1936 |
|  |  | 2 - 3 | 1.01 (0.70 – 1.46) | 0.9466 |
|  |  | 4+ | 0.94 (0.65 – 1.37) | 0.7431 |
| Conflict events | |  | 1.42 (1.26 – 1.59) | <0.0001 |
| Governorate | | Rural Damascus | Comparison |  |
|  |  | Idlib | 3.43 (2.06 – 5.71) | <0.0001 |
|  |  | Aleppo | 3.11 (1.93 – 5.00) | <0.0001 |
|  |  | Al-Hassakeh | 1.92 (1.31 – 2.81) | 0.0009 |
|  |  | Dara | 1.32 (0.85 – 2.05) | 0.2118 |
|  |  | Damascus | 0.83 (0.51 – 1.37) | 0.4690 |
|  |  | Hama | 0.73 (0.50 – 1.06) | 0.0994 |
|  |  | Homs | 0.48 (0.33 – 0.72) | 0.0003 |
|  | AIC |  | 8,751.48 | |

**Table S2**: Model 3 run with healthcare attacks in the prior 1, 2, and 3 months

**Model Fit Statistics**

AIC and deviance explained for each model are shown in **Table S3**.

| **Model Number** | **AIC** | **Deviance Explained** |
| --- | --- | --- |
| **Model 1** | 2,922.85 | 46.9% |
| **Model 2** | 21,339.74 | 55.2% |
| **Model 3** (no lag) | 8,745.30 | 74.3% |
| **Model 3** (t-1) | 8,771.07 | 73.0% |
| **Model 3** (t-3) | 8,764.69 | 73.3% |
| **Model 3** (t-6) | 8,774.26 | 72.9% |

**Table S3:** Model fit statistics for each model in the study
